# Chronic metformin treatment decreases cardiac injury during ischemia-reperfusion by attenuating endoplasmic reticulum stress with improved mitochondrial function

**DOI:** 10.18632/aging.202858

**Published:** 2021-03-22

**Authors:** Qun Chen, Jeremy Thompson, Ying Hu, Edward J. Lesnefsky

**Affiliations:** 1 Departments of Medicine, Division of Cardiology, Virginia Commonwealth University, Richmond, VA 23298, USA; 2 Biochemistry and Molecular Biology, Virginia Commonwealth University, Richmond, VA 23298, USA; 3 Physiology and Biophysics, Virginia Commonwealth University, Richmond, VA 23298, USA; 4 McGuire Department of Veterans Affairs Medical Center, Richmond, VA 23249, USA

**Keywords:** electron transport chain, electron transport complex I, endoplasmic reticulum stress, myocardial infarction, mitochondrial permeability transition pore

## Abstract

Aging impairs mitochondrial function that leads to greater cardiac injury during ischemia and reperfusion. Cardiac endoplasm reticulum (ER) stress increases with age and contributes to mitochondrial dysfunction. Metformin is an anti-diabetic drug that protects cardiac mitochondria during acute ER stress. We hypothesized that metformin treatment would improve preexisting mitochondrial dysfunction in aged hearts by attenuating ER stress, followed by a decrease in cardiac injury during subsequent ischemia and reperfusion.

Male young (3 mo.) and aged mice (24 mo.) received metformin (300 mg/kg/day) dissolved in drinking water with sucrose (0.2 g/100 ml) as sweetener for two weeks versus sucrose vehicle alone. Cytosol, subsarcolemmal (SSM), and interfibrillar mitochondria (IFM) were isolated. In separate groups, cardioprotection was evaluated using *ex vivo* isolated heart perfusion with 25 min. global ischemia and 60 min. reperfusion. Infarct size was measured.

The contents of CHOP and cleaved ATF6 were decreased in metformin-treated 24 mo. mice compared to vehicle, supporting a decrease in ER stress. Metformin treatment improved OXPHOS in IFM in 24 mo. using a complex I substrate. Metformin treatment decreased infarct size following ischemia-reperfusion. Thus, metformin feeding decreased cardiac injury in aged mice during ischemia-reperfusion by improving pre-ischemic mitochondrial function via inhibition of ER stress.

## INTRODUCTION

Elders have an increased prevalence of coronary artery disease placing them at increased risk of acute myocardial infarction [1]. Despite successful guideline-based reperfusion treatment, elders nonetheless sustain larger infarcts with greater mortality from ST elevation myocardial infarction [2, 3]. The greater susceptibility to ischemia-reperfusion injury is also observed in animal models [4, 5]. The increased cardiac susceptibility with aging in experimental models is due largely to age-induced mitochondrial dysfunction [6]. Oxidative phosphorylation (OXPHOS) is decreased in aged heart mitochondria due to impairment of the mitochondrial respiratory chain [7–10]. The dysfunctional respiratory chain increases reactive oxygen species (ROS) production [11] that sensitizes to mitochondrial permeability transition pore (MPTP) opening that in turn leads to cell death during ischemia-reperfusion [7, 12–14].

There are two populations of cardiac mitochondria that consist of subsarcolemmal mitochondria (SSM) and interfibrillar mitochondria (IFM). SSM exist underneath the sarcolemmal membrane whereas IFM are found between myofibrils and in the perinuclear region [7, 15, 16]. Mitochondrial defects mainly occur in IFM during aging [7, 17]. Contributing mechanisms of mitochondrial dysfunction with age include oxidative modifications and deletions in mitochondrial DNA [18], oxidative modification of proteins [19], activation of mitochondrial proteases [20], the impaired removal of damaged mitochondria via mitophagy [21, 22] and an increase in endoplasmic reticulum stress [17]. Many of these mechanisms imply that aging-mediated mitochondrial dysfunction is irreversible. Previous work from our laboratory supported the intriguing finding that the defects in aged heart mitochondria can be reversed [6, 17]. Furthermore, treatment of animals during the progression of aging can lead to the attenuation of age-related cardiac mitochondrial dysfunction [8]. Thus, the aged heart is not condemned to sustain greater injury due to the presence of dysfunctional mitochondria. Treatment of aged rats with the small molecule metabolite acetylcarnitine in the baseline condition improved mitochondrial function [6], supporting that the aging-induced mitochondrial defect is potentially reversible. Following an improvement in the previously established age-related mitochondrial dysfunction, aged rat hearts sustained substantially less injury during a subsequent ischemia-reperfusion stress [6]. These results indicate that reversing mitochondrial defects in aged hearts is possible and could be a promising strategy to attenuate cardiac injury during ischemia and reperfusion [6, 23].

Mitochondria are in close contact with the endoplasmic reticulum (ER) [24, 25]. ER dysfunction (ER stress) is one of the factors that induces mitochondrial defects in adult hearts [26, 27]. Induction of acute ER stress using thapsigargin (a calcium-ATPase inhibitor) impairs mitochondrial function in adult hearts as shown by decreased oxidative phosphorylation and decreased respiratory enzyme activities [26, 28], and a greater sensitization to MPTP opening [17, 26, 27, 29]. Interestingly, aging leads to increased ER stress that occurs earlier than the onset of mitochondrial dysfunction during aging [17]. These results suggest that the ER stress leads to mitochondrial dysfunction in aged hearts [17].

Metformin, an anti-diabetic drug, reduces heart injury during ischemia-reperfusion by activating AMP-activated protein kinase (AMPK) signaling [26, 30, 31]. The activation of AMPK reduces cell injury during oxidative stress via decreased MPTP opening [26, 32]. Treatment with metformin in higher dose decreases cardiac injury through inhibition of complex I during early reperfusion [33]. Metformin treatment also decreases ER stress. Angiotensin II-induced ER stress is decreased with metformin treatment by activating AMPK [26, 34]. Metformin decreases β-cell lipotoxicity by a decrease in ER stress [35]. Metformin treatment improved mitochondrial function following thapsigargin-induced acute ER stress [26]. Thus, we tested the hypothesis that preexisting ER stress in the aged hearts can be attenuated with chronic metformin treatment. If ER stress was indeed decreased, then the contribution of a reduction in ER stress to an improvement in the preexisting age-induced cardiac mitochondrial dysfunction was evaluated. Finally, the relationship of the reversal of mitochondrial defects in aged hearts to the extent of cardiac injury during subsequent ischemia and reperfusion in the high-risk aged heart was challenged.

## RESULTS

### Metformin increased the phosphorylation of AMPK in aged hearts

**Figure 1:**
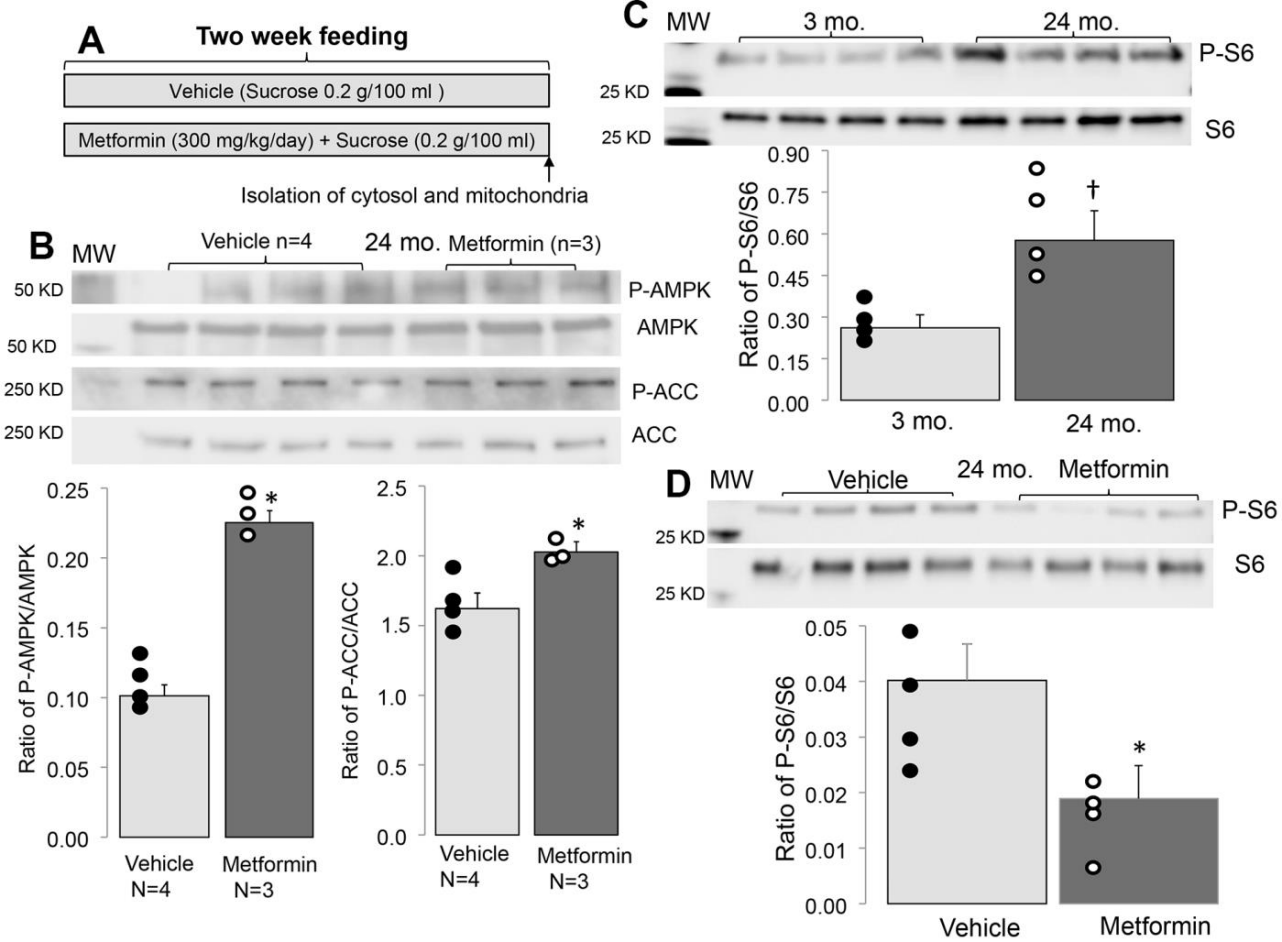
**Administration of metformin increased phosphorylation of AMPK and ACC in aged mouse hearts.** (**A**) Shows the protocol of metformin feeding. In metformin treated groups, metformin (300 mg/kg/day body weight) was dissolved in drinking water with sucrose (0.2g/100 ml) as sweetener and fed to mice for 2 weeks. In vehicle treated groups, mice were fed with drinking water with added sucrose (0.2g/100 ml). Compared to vehicle, metformin treatment increased the phosphorylation of AMPK and ACC in aged hearts, supporting that metformin feeding activates the AMPK in the aged hearts (**B**). The phosphorylation of protein S6 was increased with age, indicating an increased activity of mTORC1 (**C**). Metformin treatment decreased the age induced S6 phosphorylation (**D**). Mean ± SEM. *p<0.05 vs. vehicle, †p<0.05 vs. 3 mo.

Metformin treatment increases the phosphorylation of AMPK (Thr172) in adult mice compared to vehicle [26]. Compared to vehicle treatment, metformin treatment increased the phosphorylation of AMPK and acetyl-CoA carboxylase (Ser70) (ACC) in 24 mo. hearts (Figure 1B). ACC is phosphorylated by activated AMPK and is an index of functional AMPK activation [31]. Thus, metformin treatment also activates AMPK in the aged hearts [26].

### Metformin decreased mTOR activation in 24 mo. mice

The AMPK activation by metformin decreases the activity of mTOR (mechanistic target of rapamycin activity) [26, 36]. mTOR includes two complexes: one is mTORC1 (mTOR complex 1), and the second is mTORC2 (mTOR complex 2) [37]. Since mTORC1 is linked to ER stress by the unfolded protein response [38], the mTORC1 activation state was assessed using the phosphorylation state of ribosomal protein S6 in 24 mo. mice [17]. Compared to 3 mo., phosphorylated S6 content was significantly increased in 24 mo. hearts (Figure 1C). Metformin treatment decreased the phosphorylation of S6 in 24 mo. mice compared to vehicle (Figure 1D), suggesting that metformin treatment leads to mTORC1 inhibition in 24 mo. mice.

### Metformin decreased ER stress in the aged hearts

**Figure 2:**
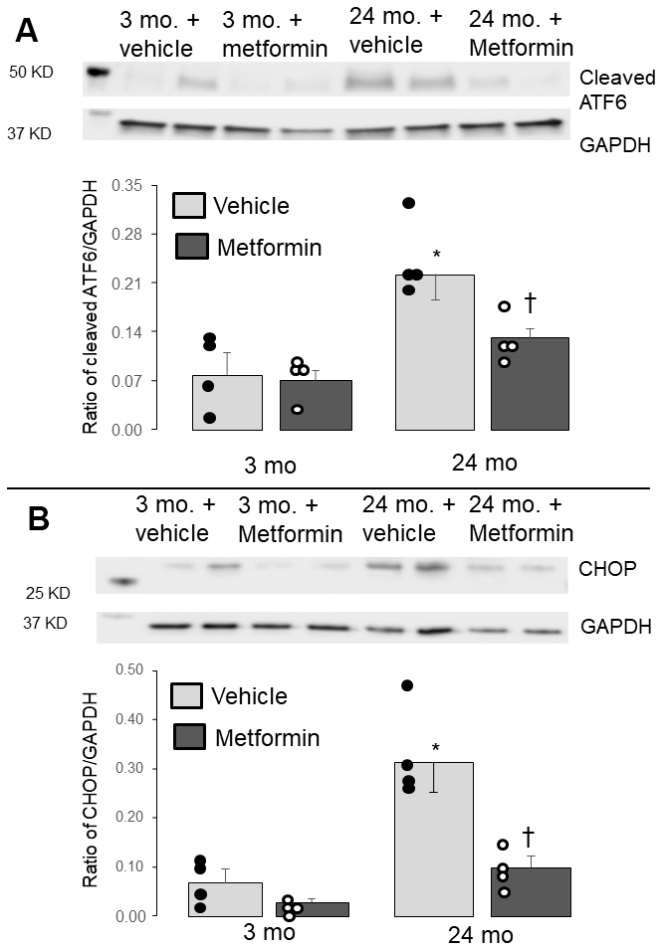
**Administration of metformin decreased the endoplasmic reticulum (ER) stress in aged mouse hearts.** Compared to 3 mo., the contents of the cleaved ATF6 (**A**) and CHOP (**B**) were significantly increased in 24 mo., supporting the presence of increased ER stress in aged hearts. The contents of cleaved ATF6 and CHOP were markedly decreased in metformin-treated 24 mo. hearts compared to vehicle, supporting that metformin treatment decreased the ER stress present in aged hearts. Metformin treatment did not alter the ER stress in 3 mo. hearts. Mean ± SEM. *p<0.05 vs. 3 mo. vehicle, †p<0.05 vs. 24 mo. vehicle. n=4 in each group.

The induction of ER stress increased CCAAT/enhancer-binding protein homologous protein (CHOP) content and the cleavage of ATF6 (cleaved activated transcription factor 6) [17, 39]. Therefore, CHOP and cleaved ATF6 are a robust indicator of ER stress [17, 39]. The contents of the cleaved ATF6 and CHOP were elevated in 24 mo. versus 3 mo. mice (Figure 2A, 2B), indicating an increased ER stress in aged hearts. Metformin treatment decreased the contents of cleaved ATF6 and CHOP in 24 mo. hearts (Figure 2A, 2B), supporting that metformin treatment decreases the ER stress that occurs during aging.

### Metformin improved oxidative phosphorylation in aged IFM

Aging leads to electron transport chain defects in IFM [40]. Compared to 3 mo., the rate of state 3 respiration in IFM was decreased in 24 mo. in the presence of complex I substrates (glutamate + malate) (Table 1) [17]. The maximal rate of ADP-stimulated respiration was also decreased in IFM from 24 mo. (Table 1). The ADP-limited respiration (state 4) in IFM was also slightly decreased in 24 mo. (Table 1). The uncoupled respiration stimulated with dinitrophenol was decreased in 24 mo. IFM (Figure 1C), localizing the defect to the electron transport chain [41].

**Table 1:** The rate of oxidative phosphorylation in IFM from young and aged mice with or without metformin treatment.

**Mice**	**3 mo.**		**24 mo.**	
Groups	Vehicle (n=13)	Metformin (n=5)	Vehicle (n=11)	Metformin (n=5)
	Complex I substrates: glutamate + malate			
State 3 (nAO/min/mg)	356±13	367±14	271±13*	337±22†
State 4 (nAO/min/mg)	58±3	58±5	48±1*	58±3†
RCR	6.3±0.2	6.6±0.5	5.7±0.2	5.9±0.5
2 mM ADP (nAO/min/mg)	481±17	487±29	318±19*	397±32†
	Complex II substrates: Succinate + rotenone			
State 3 (nAO/min/mg)	860±30	735±13*	779±34	847±30
State 4 (nAO/min/mg)	256±12	226±11	212±8*	218±8
RCR	3.4±0.1	3.3±0.1	3.7±0.1	3.9±0.1
2 mM ADP (nAO/min/mg)	849±29	746±19*	769±34	788±32

Mean ± SEM. *p<0.05 vs. 3 mo. Vehicle, †p<0.05 vs. 24 mo. Vehicle.

The state 3 respiration in IFM was not decreased in 24 mo. with succinate as complex II substrate using rotenone to block potential reverse electron flow (Table 1) [42, 43]. The rate of state 4 respiration in IFM was decreased in 24 mo. using succinate (Table 1). The high ADP-stimulated respiration was not decreased in 24 mo. IFM compared to 3 mo. with complex II substrates (Table 1), suggesting that the maximal rate of respiration with complex II substrate was not altered in aged IFM. These results localize the electron transport defect with aging predominantly to complex I in murine IFM [17].

**Figure 3:**
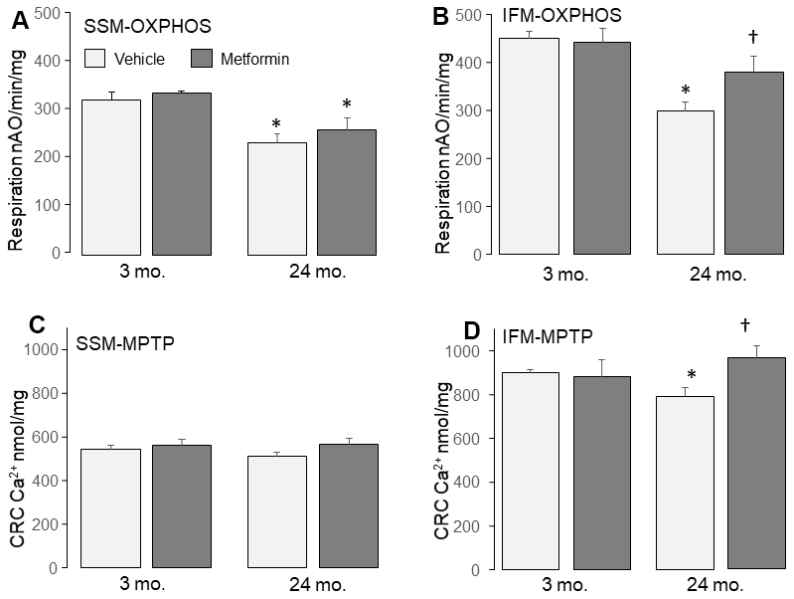
**Administration of metformin improved mitochondrial function in aged IFM.** Compared to 3 mo., dinitrophenol (DNP) uncoupled respiration was decreased in 24 mo. SSM (**A**) and IFM (**B**) using complex I substrate, supporting that aging impairs the mitochondrial respiratory chain. Metformin feeding improved oxidative phosphorylation in 24 mo. IFM oxidizing complex I substrates (**B**). Metformin feeding did not affect the oxidative phosphorylation in 24 mo. SSM with complex I substrates (**A**). Compared to 3 mo., the calcium retention capacity (CRC) was decreased in 24 mo. IFM (**D**), supporting that aging sensitizes to mitochondrial permeability transition pore (MPTP) opening. Metformin feeding improved the CRC in 24 mo. IFM (**D**) but not in 24 mo. SSM (**C**), indicating that metformin feeding decreased MPTP opening in 24 mo. IFM. Mean ± SEM; * *p* <0.05 vs. 3 mo. vehicle. †p<0.05 vs. 24 mo. vehicle. n=13 in 3 mo. vehicle group. N=5 in 3 mo. metformin treatment group. n=10 in 24 mo. vehicle group. N=9 in 24 mo. metformin treatment group.

Metformin treatment improved state 3 respiration in 24 mo. IFM in the presence of either complex I or II substrates (Table 1). Metformin treatment also improved the dinitrophenol-uncoupled respiration in 24 mo. IFM with complex I substrate (Figure 3B), supporting an improvement in the age-induced defect in electron transport. Metformin also increased state 4 respiration in 24 mo. IFM (Table 1). Importantly, MET did not alter mitochondrial respiration in 3 mo. mice without a defect in OXPHOS.

### Metformin decreased the age-induced MPTP opening in IFM

A decrease in calcium retention capacity (CRC) is an indicator of an increased sensitivity to MPTP opening in mitochondria [26, 44]. The CRC was significantly decreased in 24 mo. IFM compared to 3 mo. (Figure 3D), supporting that aging sensitizes to MPTP opening in IFM. Metformin led to a decreased sensitivity to MPTP opening in IFM from 24 mo. hearts (Figure 3D). These results indicate that metformin treatment lessens the probability of MPTP opening in aged IFM.

### Metformin did not improve oxidative phosphorylation in aged SSM with a minor age-related defect in respiration

The IFM are the dominant site of age-induced defects in mitochondrial respiration [7, 9, 40]. However, with age, SSM also exhibit a minor decrease in respiration [45]. Compared to adult, state 3 respiration in SSM was decreased in aged hearts with complex I substrate (Table 2). However, the state 4 respiration and respiratory control ratio (RCR) were not changed. (Table 2). The high ADP-stimulated respiration [46] (Table 2) and the uncoupled respiration (Figure 1B) were also decreased in SSM at 24 mo. versus 3 mo. The state 3 respiration in SSM was also decreased in 24 mo. with succinate as substrate whereas the state 4 respiration was unchanged (Table 2). Maximal ADP-stimulated respiration was not decreased in 24 mo. with complex II substrate (Table 2), indicating relative intact of complex II in aged SSM.

**Table 2:** The rate of oxidative phosphorylation in SSM from young and aged mice with or without metformin treatment.

**Mice**	**3 mo.**		**24 mo.**	
Groups	Vehicle (n=13)	Metformin (n=5)	Vehicle (n=11)	Metformin (n=5)
	Complex I substrates: glutamate + malate			
State 3 (nAO/min/mg)	291±16	326±7	220±14*	243±13
State 4 (nAO/min/mg)	44±4	48±3	37±2	41±2
RCR	6.9±0.4	7.1±0.5	6.1±0.3	6.1±0.6
2 mM ADP (nAO/min/mg)	345±16	377±6	251±21*	272±25
	Complex II substrates: Succinate + rotenone			
State 3 (nAO/min/mg)	642±25	587±24	557±20*	599±30
State 4 (nAO/min/mg)	179±8	172±8	149±5*	156±6
RCR	3.6±0.1	3.4±0.1	3.7±0.1	3.9±0.2
2 mM ADP (nAO/min/mg)	593±25	529±22	532±21	545±23

Mean ± SEM. *p<0.05 vs. 3 mo. Vehicle.

Metformin treatment did not improve OXPHOS in SSM from either 3 or 24 mo. (Table 2 and Figure 3A). The CRC was not altered by age in SSM (Figure 3C) supporting that age did not increase the sensitivity to MPTP opening in SSM in the baseline state (Figure 3C).

### Metformin decreased cardiac injury following ischemia-reperfusion

**Figure 4:**
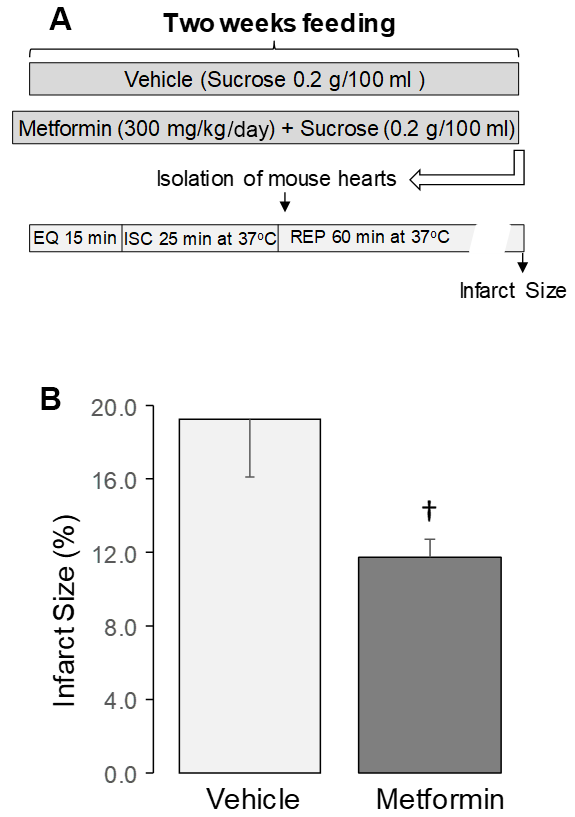
**Administration of metformin decreased cardiac injury in aged mouse hearts.** (**A**) Shows the experimental protocol. Metformin feeding was the same as in Figure 1. Isolated perfused hearts received no *ex vivo* treatment. The isolated mouse hearts underwent 25 min. global ischemia at 37° C and 60 min. reperfusion. Metformin treatment decreased the infarct size in aged 24 mo. hearts compared to vehicle (**B**), supporting that metformin treatment decreased cardiac injury in 24 mo. hearts following ischemia-reperfusion. Mean ± SEM. †p<0.05 vs. 24 mo. vehicle. n=9 in vehicle treated group. n=10 in metformin treated group.

Metformin feeding did not affect heart or body weight in 24 mo. old mice (Table 3). Metformin treatment did not alter left ventricular developed pressure (LVDP) [47] (Table 3) or end diastolic pressure (LVEDP) (Table 3) before ischemia in 24 mo. hearts compared to vehicle. Ischemia followed by reperfusion led to decreased LVDP and increased LVEDP in both vehicle and metformin-treated hearts compared to the pre-ischemia value (Table 3). However, metformin treatment markedly decreased the infarct size compared to vehicle (Figure 4B), supporting that metformin treatment that leads to the restoration of mitochondrial function mitigates cardiac injury in aged hearts during subsequent *in vitro* ischemia and reperfusion.

**Table 3:** Hemodynamic change during ischemia-reperfusion in 24 mo. hearts with or without metformin treatment.

	**Vehicle (n=9)**	**Metformin (n=10)**
Body weight (g)	33.1 ± 1.5	33.6 ± 1.2
Heart weight (g)	0.17 ± 0.01	0.18 ± 0.01
Ratio of Heart/body	0.0050 ± 0.0003	0.0053 ± 0.002
	Pre-Ischemia	
LVDP (mmHg)	82 ± 8	73 ± 2
LVEDP (mmHg)	5 ± 1	6 ± 1
	End of Reperfusion	
LVDP (mmHg)	39 ± 5*	30 ± 7*
LVEDP (mmHg)	32 ± 6*	34 ± 7*

Mean ± SEM. *p<0.05 vs. corresponding pre-ischemic value. All p=NS vehicle vs. metformin.

## DISCUSSION

In the present study, we show that chronic treatment for two weeks with metformin improved pre-existing age-induced ER stress (Figure 2) and mitochondrial dysfunction (Figure 3B). Next, we asked if the relief of ER stress with improved mitochondrial function led to decreased cardiac injury following ischemia and reperfusion. In the buffer perfused aged heart, infarct size was substantially reduced (Figure 4D). As expected, metformin feeding increased AMPK activation as shown by the phosphorylation of both AMPK and ACC (Figure 1). Thus, a translational relevant approach can attenuate metabolic defects present in the aged heart and reduce injury in the aged heart from superimposed cardiac stress [41]. Furthermore, this treatment approach uses the novel approach to decrease cardiac injury from ischemia and reperfusion by the treatment of preexisting age-induced disease.

OXPHOS is mainly impaired in IFM from aged hearts [7]. A decrease in dinitrophenol-uncoupled respiration supports that the defects in aged heart mitochondria are located in the electron transport chain [7, 40, 48]. Aging also sensitizes to MPTP opening mainly in IFM [14]. The results support previous findings that the aging defect predominantly involves IFM. The ER stress increases in aged hearts [17, 49]. Metformin attenuates the ER stress in aged hearts indicated by decreased CHOP and cleaved ATF6 contents (Figure 2). CHOP is a major endpoint of the activation of ER stress, with increased formation not only via cleavage of ATF6, but also via phosphorylation and activation of PERK (RNA-activated protein kinase-like ER kinase) [50] as well as the activation of IRE1α (inositol requiring enzyme 1) [51–55]. Concomitant with the reduction in ER stress, metformin treatment improves OXPHOS and decreases the sensitivity of MPTP opening in aged IFM (Figure 3D). These results support that the increased ER stress causes mitochondrial dysfunction in the aged heart [17]. Restoration of mitochondrial function prior to the onset of ischemia and reperfusion in aged hearts by metformin, in turn, decreases cardiac injury during ischemia-reperfusion. Thus, chronic metformin treatment could be a promising strategy to protect aged hearts during ischemia and reperfusion by reducing the ER stress present in the baseline state.

Chronic metformin pretreatment decreases infarct size in adult mice [56]. Furthermore, ongoing metformin treatment appears beneficial in diabetic patients who suffer a subsequent infarct [57]. Metformin treatment in diabetic patients substantially improves cardiovascular disease outcomes in the UK Prospective Diabetes Study (UKPDS) [58]. Metformin protects in experimental models of heart failure [57]. Metformin is well tolerated in non-diabetic mice [33, 59]. The GIPS-III trial found that metformin therapy was also safe in non-diabetic adult patients, including those suffering an acute myocardial infarction [60]. Thus, there is a substantial potential for a repurposing in elders to attenuate age-induced susceptibility of the heart.

The mechanisms by which aging leads to mitochondrial dysfunction remain unclear. In the present study, we find that ER stress is markedly elevated in aged hearts. In addition, the reduction of ER stress using metformin improves mitochondrial function in aged hearts. The results support that the increased ER stress causes mitochondrial dysfunction during aging. Metformin treatment decreased ER stress in other disease models including catecholamine stress [61], pressure overload [34] and diabetes [62]. However, the mechanisms of action of metformin from these studies also remains to be better defined. Metformin may decrease ER stress due to modulation of SERCA activity in the adult heart [26, 63]. To our knowledge, the improvement in mitochondrial function in the aged heart with attenuation of the ER stress by metformin therapy has not been previously described.

Two weeks of metformin therapy markedly reduced ER stress and subsequent mitochondrial dysfunction in the adult heart [26, 28]. This previous work from our laboratory [26] demonstrates that metformin decreases the thapsigargin-induced ER stress in adult hearts with the protection of mitochondrial oxidative phosphorylation and complex I of the respiratory chain [26, 28]. In the present study, metformin can decrease the age-induced pre-existing ER stress present in aged hearts before the occurrence of other cardiac disease. These results support that metformin is an option to decrease the ER stress in aged hearts before the onset of a superimposed stress such as ischemia and reperfusion. Administration of the AMPK activator 5-aminoimidazole-4-carboxyamide-1-beta-D-ribofuranoside decreases the ER stress in cardiac myocytes during hypoxia-reoxygenation [64], indicating that activation of AMPK decreases the ER stress. Metformin is clearly a more translational relevant approach to modulate ER stress and improve mitochondrial function than the classic small molecule chaperone 4-phenylbutyrate (4-PBA) that is commonly used to inhibit ER stress in animal studies [65], including during aging [17, 66, 67]. To further support the role of age-induced ER stress in mitochondrial dysfunction during aging, previous work showed that two weeks of treatment with the 4-PBA reduced ER stress and improved pre-existing age-induced mitochondrial dysfunction [17, 68].

Metformin is a traditional anti-diabetic drug with cardioprotective effects by activating AMPK signaling [69]. The mechanism of AMPK activation is due to subtle complex I inhibition, leading to a modest decrease in energy charge. This inhibition is observed with chronic metformin therapy and requires only micromolar intracellular concentration [70, 71]. Metformin, via this AMPK activation, leads to enhanced mitophagy and mitochondrial biogenesis [72–74]. Metformin downregulates the mechanistic target of rapamycin through activation of AMPK [36, 75]. In addition to the relief of ER stress, the potential mechanisms of AMPK protection include favoring glucose uptake and oxidation, modulation of autophagy [76], and augmentation of mitochondrial biogenesis [77]. In the present study, chronic metformin treatment activates AMPK in aged hearts as shown by increased phosphorylation of the AMPK and ACC. These results suggest that metformin decreases the ER stress during aging by activating the AMPK signaling. Interestingly, a recent study shows that the decreased PGC1-α level in aged hearts is not dependent on AMPK activity. Metformin treatment does improve mitochondrial biogenesis in aged hearts [78]. These results suggest that metformin-mediated improvement in biogenesis is not solely dependent on AMPK activation.

Activation of AMPK by metformin leads to decreased mTOR activity through activation of AMPK [36, 37]. The mTORC1 performs the classic functions of mTOR including nutrient sensing, regulation of protein synthesis, and autophagy [37], whereas the mTORC2 is involved in cell proliferation and insulin signaling [37]. mTORC1 is linked to ER stress via the unfolded protein response [38]. Inhibition of mTOR extends lifespan [79]. mTORC1 regulates the translation of several mitochondria-related mRNAs including components of complex I and V and transcription factor A (TFAM) [80]. Inhibition of mTORC1 in 24 mo. mice improves cardiovascular function and reverses cardiac fibrosis [81] by improving mitochondrial function [82]. The inhibition of mTORC1 by activation of AMPK itself prolongs lifespan [82, 83]. The current study shows that metformin treatment results in activation of AMPK in 24 mo. old mice accompanied by decreased mTORC1 activation indicated by decreased phosphorylation of the protein S6. These results support the notion that activation of AMPK provides protective benefits in aged hearts by inhibiting mTORC1.

AMPK activation is enhanced by the sestrin protein family [84]. Sestrin2 is a stress-induced scaffold protein that mediates AMPK activation via interaction with LKB1 [5, 85, 86]. Sesn2 expression at baseline was reduced in aged hearts [5, 86] and a decrease in Sesn2 attenuates AMPK activation. Sesn2 mediated AMPK activation also leads to the downregulation of mTOR [87, 88] that can protect cells against ER stress by decreasing protein synthesis [89, 90]. The age-induced decrease in Sesn2 expression may impair activation of AMPK in response to stress. However, in the current study, chronic metformin therapy was nonetheless able to activate AMPK to a sufficient extent to reduce ER stress and improve mitochondrial function, similar to protection against ER stress in the adult heart [26].

In addition to increased cardiac injury during ischemia-reperfusion, aging also invalidates endogenous signaling of cardiac protection that leads to many cardioprotective approaches that protect younger adult hearts, leading these approaches to fail in aged hearts [7, 12, 91]. Treatment of aged rats with the small molecule metabolite acetylcarnitine improved mitochondrial function [6], supporting the potential reversibility of the mitochondrial defect in aging. This concept is further supported by current findings that metformin restores mitochondrial function in the aged hearts. Treatment with acetylcarnitine in the aged heart not only restored mitochondrial function, but also reduced cardiac injury during ischemia-reperfusion [6], indicating that the age-induced mitochondrial defects contribute to cardiac injury [6]. The current study shows that metformin treatment improves mitochondrial function and decreases cardiac injury in aged hearts during ischemia and reperfusion, expanding available options to restore mitochondrial function in aged hearts. Metformin treatment may provide additional benefits in the high-risk elderly population with a greater incidence of myocardial infarction [2, 3] that suffer substantially greater cardiac injury and decreased survival should an infarction occur [2, 3].

MPTP opening leads to cell death during ischemia and reperfusion [92]. Increased ER stress favors MPTP opening in adult heart mitochondria [26–28]. In the present study, aging leads to increased sensitivity to MPTP opening mainly in IFM. Importantly, attenuation of the ER stress with metformin decreases MPTP opening in mitochondria isolated from aged hearts. The current study provides direct evidence that the increased ER stress favors MPTP opening during aging. ER stress mediated oxidative [93] and calcium-driven [27] mechanisms impact mitochondria and likely contribute to the increased susceptibility to MPTP opening even in the baseline state. Metformin treatment decreases the MPTP opening during acute ER stress by activating AMPK [26]. This mechanism may be also involved in decreased MPTP opening in aged hearts with metformin treatment.

### Limitations

The current study emphasized a translational relevant approach to activate AMPK driven reduction in ER stress in the baseline aging condition to improve mitochondrial function. The response from aged hearts paralleled the response of metformin therapy to reduce ER stress, likely via AMPK mediated mechanisms. The period of pretreatment that improved mitochondrial function in the current study most certainly resulted in modest inhibition of complex I that was present during ischemia and reperfusion, even in the absence of additional metformin treatment [70, 71] which would have exerted protection in aged heart during ischemia [94] and early reperfusion [11]. Although inhibition of complex I by metformin leads to deceased cardiac injury during ischemia-reperfusion [33], other mechanisms may be also involved in the protection of metformin. The improvement of OXPHOS, especially with complex I substrates, does not suggest that substantial complex I inhibition was present before ischemia and reperfusion from the chronic metformin treatment, though a beneficial impact regarding production of reactive oxygen species production and attenuation of permeability transition pore opening cannot be excluded [70]. Metformin may decrease cardiac injury by decreasing sensitivity to MPTP opening as shown in cultured cells and isolated mitochondria [71]. ROS generated by reverse electron flow contributes to cardiac injury during reperfusion [95]. Metformin treatment likely also mitigates cardiac injury through reduction of the reverse flow-induced ROS generation [70]. Activation was sufficient despite age-related decrements in sestrin 2 content, the role of which will also require further study. The mechanisms of the ER and mitochondrial interactions that favor age-induced mitochondrial dysfunction will require additional work. Finally, the model of ischemia and reperfusion is one of acute injury, the study of longer-term reperfusion and recovery periods, especially the *in vivo* ischemia-reperfusion model, will be required in the future.

## CONCLUSIONS

**Figure 5:**
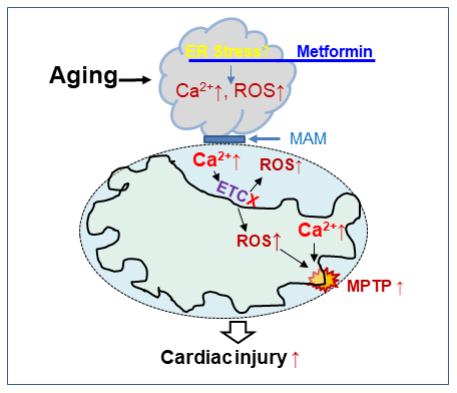
**The chronic administration of metformin decreases endoplasmic reticulum stress with improvement in cardiac mitochondrial function in aged mouse hearts.** Aging increases endoplasmic reticulum (ER) stress that causes mitochondrial dysfunction by increasing calcium overload and ROS generation. The ER and cardiac mitochondrial interact via mitochondrial associated membranes (MAM). An increase in mitochondrial calcium overload and ROS generation sensitizes to mitochondrial permeability transition pore (MPTP) opening that augments cardiac injury during ischemia-reperfusion. Metformin treatment decreases cardiac injury by restoring mitochondrial function before ischemia through attenuation of the ER stress in the aged hearts.

Chronic treatment of aged mice for two weeks with metformin activated AMPK signaling leading to a reduction in age-induced ER stress with substantial improvement of function in heart mitochondria (Figure 5). In the setting of these improvements, the age-enhanced susceptibility to cardiac injury during myocardial infarction and reperfusion was improved. The current study advances the understanding of mechanisms involved in aging-mediated mitochondrial dysfunction, but also provides a novel treatment opportunity to restore mitochondrial function in aging in that the ER stress can be modulated by pharmacologic interventions. This translational treatment approach provides key evidence for a new treatment paradigm to decrease injury from superimposed cardiac disease in the high-risk aged heart by the treatment of age-related defects to restore mitochondrial function before the onset of acute cardiac disease in the elderly heart. This approach is potentially relevant to acute coronary syndromes with ST elevation-induced myocardial infarction with an increased risk of progression to heart failure [2, 96], acute coronary syndromes with non-ST elevation infarction [97, 98], heart failure with preserved ejection fraction [99, 100], and chemotherapy cardiotoxicity [101].

## MATERIALS AND METHODS

### Metformin treatment

The Animal Care and Use Committees of Virginia Commonwealth University and the McGuire Department of Veterans Affairs Medical Center approved the study. Male mice of young adult (3 mo.) and aged mice (24 mo.) were used in this study. Mice were given a normal diet with *ad libitum* access to food and water throughout the experiment. Normal diet included 16% protein and 4% fat. In metformin treated mice, metformin (300 mg/kg/day body weight) was dissolved in drinking water with sucrose (0.2g/100 ml) as sweetener and fed to mice for 2 weeks [56] (Figure 1A). The dose of metformin was based upon previous studies in the rat [56]. Control mice received drinking water with sucrose vehicle (0.2g/100 ml). Deep anesthesia was induced in mice with pentobarbital sodium (100 mg/kg, i.p.). Then, mitochondria were isolated from the excised mouse heart.

### Isolation of cytosol, mitochondria, and nucleus

SSM and IFM were isolated as previously described [17]. The mouse heart was first placed in cold buffer A (composition in mM: 100 KCl, 50 MOPS [3-(N-morpholino) propanesulfonic acid], 1 EGTA, 5 MgSO_4_, and 1 ATP). The heart was blotted dry, weighed, and homogenized using a polytron tissue homogenizer at 10,000 rpm for 2.5 seconds. The polytron homogenate was first centrifuged at 500 *g* for 10 min. The supernatant was used to isolate SSM with further centrifugation at 3,000 g for 10 min. The pellet from the 500 g centrifuge step was washed and used for IFM isolation. The skinned myofibers, obtained from the polytron homogenization step, were resuspended in buffer A and incubated with 5 mg/g (wet weight) trypsin for 10 min at 4° C. Then, buffer B [buffer A including 0.2% bovine serum albumin (BSA)] was added to stop trypsin effect. The crude SSM and IFM were washed with buffer B. The purified SSM and IFM were suspended in 80 mM KCl, 50 mM MOPS, and 0.5 mM EGTA for functional measurement.

Oxygen consumption in isolated mitochondria was measured using a Clark-type oxygen electrode at 30° C as previously described [102]. Glutamate (20 mM) + Malate (10 mM) were used as complex I substrate. Succinate (20 mM) was used as the complex II substrate with the inclusion of 7.5 μM rotenone. ADP (2 mM) was used to determine the maximal rate of ADP-stimulated respiration.

### Calcium retention capacity (CRC) in isolated mitochondria

The CRC was used to assess the calcium induced MPTP opening in freshly isolated SSM and IFM [103]. The assay medium included mitochondria (125 μg/ml), 150 mM sucrose, 50 mM KCl, 2 mM KH_2_PO_4_, and 5 mM succinate in 20 mM Tris/HCl with pH at 7.4. Calcium Green-5N (0.5 uM, Thermo Scientific, Waltham, MA) was used to monitor extra-mitochondrial Ca^2+^ concentration with excitation and emission wavelengths set at 500 and 530 nm, respectively [103]. Sequential exogenous calcium (5 nmol/pulse) was added into cuvettes until MPTP opening occurred, shown by a burst release of calcium from mitochondria.

### Measurement of ROS in SSM and IFM

The amount of H_2_O_2_ generation in SSM and IFM was measured using Amplex red as a fluorogenic indicator in the presence of horseradish peroxidase. Freshly isolated SSM or IFM (200 μg) were incubated in chelex-treated buffer [pH 7.4 (150 mM KCl, 5 mM KH_2_PO_4_, 1 mM EGTA)] in the presence of 25 μM Amplex Red and 0.20 units/ml HRP. Glutamate + malate was used as complex I substrate, and succinate + rotenone was used as complex II substrate. Rotenone (complex I inhibitor) and antimycin A (complex III inhibitor) were used to induce maximal H_2_O_2_ generation from complex I and complex III, respectively [104].

### Isolated perfused heart model of Ischemia and Reperfusion

Mouse hearts were excised under deep anesthesia using pentobarbital sodium (100 mg/kg i.p.) and anticoagulated with heparin (1,000 IU/kg i.p.). The isolated heart is mounted in the Langendorff setup and perfused with modified Krebs-Henseleit (K-H) buffer oxygenated with 95% O_2_-5% CO_2_ through aorta. A balloon was inserted into the left ventricle to monitor cardiac function. The heart was first perfused with K-H buffer for 15 min. The hearts underwent 25 min. of global ischemia at 37° C and 60 min. of reperfusion (Figure 4A). In order to keep a constant heart rate, hearts were paced at 420 beats/min during the 15 min. equilibration period and after 10 min. of reperfusion [105]. Myocardial infarct size was measured at the end of the reperfusion using staining with triphenyl tetrazolium chloride (TTC) [106].

### Western blotting

Proteins from mitochondria or cytosol were separated using 12% or 4-15% Tris-glycine gels (Bio-Rad, Hercules, CA) and transferred to a PVDF membrane (Millipore) using semi-dry transfer (Bio-Rad). The membrane was incubated for 1 hour at room temperature in 5% (w/v) non-fat dry milk (Bio-Rad) in TBS-T buffer (10 mM Tris pH 7.5, 150 mM NaCl, 0.1% Tween-20). Then, the membrane was incubated with primary antibody overnight at 4° C. After 1 hour incubation at room temperature with a 1:10,000 dilution of HRP-conjugated anti-mouse or anti-rabbit IgG F(ab)_2_ (GE Healthcare Life Sciences, Piscataway, NJ), blots were developed using ECL Plus Western Blotting Detection Reagents (GE Healthcare Life Sciences, Piscataway, NJ) [28, 47].

### Statistical analysis

Data are expressed as the mean ± standard error [107]. Normality distribution was assessed. One-way analysis of variance (ANOVA) was used to compare the differences between groups (≥ 3 groups). The Student-Newman-Keuls test of multiple comparisons was used when a significant F value was obtained. The non-paired student test (two tails) was to compare the differences between two groups. Statistical significance was accepted when a value of *p* less than 0.05 was obtained.
